# Identification of QTLs Associated with Virulence Related Traits and Drug Resistance in *Cryptococcus neoformans*

**DOI:** 10.1534/g3.116.029595

**Published:** 2016-06-30

**Authors:** Aaron A. Vogan, Jordan Khankhet, Himeshi Samarasinghe, Jianping Xu

**Affiliations:** Department of Biology, McMaster University, Hamilton, Ontario L8S 4K1, Canada

**Keywords:** *Cryptococcus neoformans*, quantitative trait loci mapping, virulence, resistance

## Abstract

*Cryptococcus neoformans* is a basidiomycete fungus capable of causing deadly meningoenchephilitis, primarily in immunocompromised individuals. Formerly, *C. neoformans* was composed of two divergent lineages, but these have recently been elevated to species status, now *C. neoformans* (formerly *C. neoformans* var. *grubii*) and *C. deneoformans* (formerly *C. neoformans* var. *neoformans*). While both species can cause deadly infections in humans, *C. neoformans* is much more prevalent in clinical settings than *C. deneoformans*. However, the genetic factors contributing to their significant differences in virulence remain largely unknown. Quantitative trait locus (QTL) mapping is a powerful tool that can be used to identify genomic regions associated with phenotypic differences between strains. Here, we analyzed a hybrid cross between these two species and identified a total of 23 QTL, including five for melanin production, six for cell size, one for cell wall thickness, five for the frequency of capsule production, three for minimal inhibitory concentration (MIC) of fluconazole in broth, and three for MIC on solid medium. For the fluconazole resistance-associated QTL, three showed environment and/or concentration-specific effects. Our results provide a large number of candidate gene regions from which to explore the molecular bases for phenotypic differences between *C. neoformans* and *C. deneoformans*.

Human fungal pathogens are relatively unique among disease causing organisms. Unlike many bacterial and nonfungal eukaryotic pathogens, the fungal organisms that cause the largest global burden of infectious disease are not obligate pathogens, but opportunistic invaders. The species which inflict the heaviest toll on human life are from the genera *Aspergillus*, *Candida*, and *Cryptococcus*, which together cause upwards of 1.6 million life threatening infections annually (Brown *et al.* 2012). *Candida* species are generally human commensal organisms while Cryptococcal and Aspergilli species are commonly found in the environment. While these fungal infections typically infect immunocompromised individuals, some strains of *Cryptococcus* have caused infections in many apparently healthy individuals (Kidd *et al.* 2004; Chen *et al.* 2008). The details of what influences the transition from an environmental microbe to a pathogenic one are largely unknown, and *Cryptococcus* provides an ideal system to examine this question.

*Cryptococcus neoformans* is a basidiomycetous yeast capable of causing various forms of disease, including deadly meningoencephilitis (Mitchell and Perfect 1995). Recently, *C. neoformans* was divided into two separate species, *C. neoformans* (formerly *C. neoformans* var. *grubii* or serotype A) and *Cryptococcus deneoformans* (formerly *C. neoformans* var. *neoformans* or serotype D) (Hagen *et al.* 2015). *C. neoformans* most commonly causes life threatening disease and is more virulent in a mouse model (Kwon-Chung *et al.* 1992), whereas *C. deneoformans* is more likely to cause cutaneous infection (Dromer *et al.* 2007). Both species have been isolated throughout the globe, however *C. neoformans* originates in Africa (Litvintseva and Mitchell 2012), and *C. deneoformans* possibly originates in Europe (Boekhout *et al.* 2001). In addition to these differences, *C. deneoformans* appears to be more sensitive to cationic stress (Cruz *et al.* 2000) and may be more susceptible to some antifungal drugs (Dannaoui *et al.* 2006), though there are conflicting reports on this topic (Thompson *et al.* 2008).

It is estimated that the two species diverged from each other approximately 18.5 million years ago (Xu *et al.* 2000). Despite their long history of divergence, hybridization can occur readily between them and appears to be quite common in nature. Specifically, A/D hybrids are frequently isolated from both environmental and clinical settings, and in fact the holotype for *C. neoformans* was revealed to be a hybrid (Hagen *et al.* 2015). Phylogenetic analyses revealed recent and ongoing hybridizations (Xu *et al.* 2002; Litvintseva *et al.* 2007), however there is evidence of ancient hybridization events as well (Kavanaugh *et al.* 2006). Investigations into natural and laboratory hybrids have revealed a number of anomalies in the hybrids, including aneuploidy, abortive germination, and suppressed recombination (Lengeler *et al.* 2001; Sun and Xu 2007; Vogan *et al.* 2013; Vogan and Xu 2014).

It has been well documented that the production of melanin and capsule, and the ability to grow at high temperatures, are required for virulence in *Cryptococcus* (Wang *et al.* 1995; Kwon-Chung and Rhodes 1986; Steenbergen *et al.* 2001; Odom *et al.* 1997). Additionally, mating type may contribute significantly to the severity of infection (Barchiesi *et al.* 2005), with the specifics differing between the species (Chang *et al.* 2000; Yue *et al.* 1999; Wang *et al.* 2002). Since both species possess and require the same virulence factors, the presence of these conserved virulence factors do little to explain the different clinical prevalence of the two species. However, these two species may differ quantitatively in these traits. Thus, understanding the phenotypic differences between strains of the two species could help reveal the genetic bases for their differences in pathogenicity and contribute significantly to our understanding of the evolution of these pathogens. Quantitative trait locus (QTL) mapping is a powerful approach for identifying the genomic regions that contribute to a phenotype of interest. For example, QTL mapping has been used to elucidate the genetics of pathogen resistance in crops and, in addition, has been successfully applied to the study of the pathogens themselves (Lin *et al.* 2006; Lind *et al.* 2007; Li *et al.* 2011; Morrison *et al.* 2009; Christians *et al.* 2011; Saeij *et al.* 2006).

Previously, we conducted a cross between *C. neoformans* and *C. deneoformans*, and collected 230 hybrid offspring. For this work, we have genotyped the progeny using 73 codominant markers and constructed a linkage map. We have measured quantitative traits for the known virulence factors of melanin and capsule production, as well as cell wall thickness and cell size. Since the two parental strains differ in their susceptibility toward the antifungal drug fluconazole, we have also evaluated the growth of the progeny at a variety of fluconazole concentrations on both solid agar plates and in liquid media. The objective of this study is to identify genomic regions that contribute to the differences in these virulence-associated traits between *C. neoformans* and *C. deneoformans*.

## Materials and Methods

### Mapping population

Parental strains JEC20 (*C. deneoformans*, MATa) and CDC15 (*C. neoformans*, MATα) were mated on V8 agar for ∼4 wk. Basidiospores were dissected onto yeast extract peptone dextrose (YEPD) agar using a Singer MSM 300 micromanipulator. Basidiospores were allowed to germinate over the following 2 wk at room temperature. 230 colonies were collected to generate the mapping population in this study (Vogan *et al.* 2013; Vogan and Xu 2014).

### Melanin production

Strains were grown on YEPD agar for 2 d to 7 d at 30°. Fresh cells were collected to generate cell suspensions with $∼1\times 10^{6}$ cells/ml. Suspensions were then inoculated onto caffeic acid agar (Hopfer and Blank 1975) with four replicates per progeny per plate and allowed to grow for 3 d at 30°. Melanin production was approximated by light emission using the Spot Densitometry function of a FluoroChem 8900. Darker colonies with more melanin reflect less light than lighter colonies. To minimize batch effects, parental strains were used as references in each of our batch assays to help standardize the data for analyses.

### Cell size, cell wall, and capsule measurements

Strains were grown in Sabouraud dextrose (SD) broth for 1 d at 30°. 10 μl of the liquid culture were diluted into 190 μl of 10% SD [inducing media (Liu *et al.* 2008)], buffered with 0.165 M 3-Morpholinopropane-1-sulfonic acid (MOPS) to pH 7.3 and allowed to grow for 2 d. Slides were made with 5 μl of cell suspension and 9 μl of nigrosin counterstain (in lieu of india ink). Images were taken at 1600× magnification on an Olympus IX81 Microscope. Measurements of cell area, cell wall area, and capsule were made by manually fitting ellipses to the cells using the software ImageJ (Abramoff *et al.* 2004). Approximately 25 cells were measured per strain.

### Fluconazole assays

Strains were grown on YEPD agar for 2 d at 30° and then transferred to either YEPD agar or Roswell Park Memorial Institute (RPMI) media for solid and liquid assays, respectively. Concentrations of 0 μg/ml, 0.5 μg/ml, 1.0 μg/ml, 2.0 μg/ml, 4.0 μg/ml, 8.0 μg/ml, 16.0 μg/ml, 32.0 μg/ml, and 64.0 μg/ml of fluconazole were evaluated. For solid medium assays, fluconazole was filter sterilized and added to media prior to pouring of the plates. Growth was determined by measuring the diameter of ∼10 colonies per strain after 2 d of growth at 30°. For liquid medium assays, strains were grown in 200 μl of RPMI in 96-well plates. The OD ${}_{600}$ of wells was measured at the beginning of the experiment and at 72 hr, the difference was taken to determine growth over this time period. The minimal inhibitory concentration (MIC) for growth on solid medium was determined as the minimum concentration of fluconazole at which no colonies were observed by the naked eye. The MIC for growth in broth was determined as the concentration of fluconazole where a 90% reduction in OD ${}_{600}$ (as compared to a control with no drug) was observed. An additional concentration of 128 μg/ml was evaluated for this purpose due to a high degree of fluconazole resistance.

### Genotyping

DNA was extracted from strains using standard chloroform-isoamyl alcohol methodology (Xu *et al.* 2000). Seventy-three codominant Polymerase Chain Reaction-Restriction Length Polymorphism (PCR-RFLP) markers were used to determine genotypes for all progeny. Markers were either taken from Sun and Xu (2007), Vogan *et al.* (2013), or Vogan and Xu (2014), or designed using Prifi (Fredslund *et al.* 2005) (Supplemental Material, Table S1).

### Statistical analyses of traits

Differences in trait values between JEC20 and CDC15 were evaluated with the two-tailed Student’s t–test. Normality of traits of the progeny were evaluated with the Shapiro–Wilk test. All pairwise combinations of traits were analyzed for correlation by calculating Spearman’s Rho. Broad-sense heritability was estimated for melanin production, cell wall thickness, cell size, and MIC on agar using ANOVA based on data from all the progeny and both parental strains. However, heritability could not be calculated for capsule production, as it is a ratio in each strain, nor for MIC in broth as there was only one data point for each progeny.

### QTL analysis

The R package R/qtl was utilized for all QTL analyses (Broman *et al.* 2003). A linkage map was created by pinning markers to their physical locations within the 14 chromosomes of the reference genome of JEC21 (Loftus *et al.* 2005). A previous hybrid linkage map had been constructed by Sun and Xu (2009) which showed large scale congruence in the position of markers between both JEC21 and JEC20, and between JEC21 and H99. That study also confirmed a large scale translocation between chromosomes 8 and 12 (Sun and Xu 2009). The notations for the chromosomes used in this study correspond to the original nomenclature for *C. neoformans*, and so chromosome 1 is stated as Chromosome A, chromosome 2 is Chromosome B, and so forth. Only one marker from this study lies in the aforementioned translocated region. This marker is placed in its own linkage group “H2”.

Multiple interval mapping (MIM) was used to evaluate the presence of QTL using the stepwise function of R/qtl. This methodology searches for the QTL with the highest LOD score and then sequentially adds additional QTL that either have large effects on their own, or as interactive effects with QTL that have already been incorporated into the model. A penalized LOD score is utilized to avoid overfitting the model (Broman and Sen 2009). Haley–Knott regression was employed with a step size of 0.2 cM to compute the LOD scores between the markers; these are referred to as pseudomarkers (Broman and Sen 2009). A LOD significance threshold of α = 0.05 was determined using 1000 permutations.

A slightly different approach, multiple QTL mapping (MQM), was used to evaluate QTL for specific concentrations of fluconazole. MQM allows for the identification of multiple QTL in one analysis, whereas MIM can only evaluate one QTL at a time. This allows for rapid detection of QTL across a large number of phenotypes and can be used to identify pleiotropic effects among the examined fluconazole concentrations (Arends *et al.* 2010). Cofactor significance was set to 0.002 (as recommended in the manual for conservative analysis) and LOD threshold values were calculated with 1000 permutations.

For both MIM and MQM, models were built using the commands fitqtl and refineqtl to refine the positions of QTL and determine their significance to the model, as well as the percent of phenotypic variance that the models explain. The 1.5-LOD support intervals for the QTL were determined with the lodint function of R/qtl. Composite interval mapping (CIM) was also explored and found to be largely in agreement with the results from MIM. Phenotypic, genotypic, and map distances are found in Table S2 and Table S3. Full results from the modeling can be found in Table S4.

### Data availability

The authors state that all data necessary for confirming the conclusions presented in the article are represented fully within the article.

## Results

### Linkage map

Six or fewer markers were used to genotype each chromosome with the exception of the largest chromosome A, which was genotyped with 25 markers. The average spacing between markers was 5.6 cM and the largest spacing between any two markers on the same chromosome was 18.1 cM between marker CND05120 and RUM1. The markers used here cover 17.1Mb, accounting for about 90% of the whole genome. The entire linkage map was 322.5 cM. The ratio of cM to kb for the entire linkage map was 53.0 kb/cM. The average cM/kb ratio per chromosome was 63.5 kb/cM with a maximum ratio of 101.0 kb/cM for chromosome L and a minimum ratio of 34.0 kb/cM for chromosome A.

### QTL mapping

All phenotypes were shown to be quantitative traits with the exception of capsule size/thickness. Neither JEC20 nor CDC15 produced any capsule under the inducing conditions used here. There was a range of variation among the progeny, with some strains producing vast quantities of capsule while others produced relatively little or none. In addition, there was also variation among cells within individual strains, with some cells producing ample amounts of capsule while other cells of that strain produced none. Due to the large variations among cells within an individual strain, when capsule size was used as a trait for analysis, no QTL were identified. However, when the frequency of capsule production (based on the proportion of cells that produced capsule for a given strain) was used, multiple QTL were identified.

Broad-sense heritability for the traits was estimated at 0.98 for melanin, 0.95 for cell size, 0.91 for cell wall thickness, and 1.0 for MIC on agar. None of the traits exhibited a normal distribution according to the Shapiro–Wilk test. Interestingly, there was abundant evidence for statistically significant correlations among most of the traits (Table S5). For example, except for cell wall thickness, all traits were positively correlated with cell size. All other pairwise combinations showed significant positive correlation with each other, except for MIC in broth and melanin production, both of which were slightly negatively correlated to cell wall thickness (Table S5).

At least one QTL was found for each phenotype assayed. A summary of all QTL identified in this study are listed in Table 1. Five QTL were identified for melanin production, six for cell size, one for cell wall thickness, five for the frequency of capsule production, three for MIC in broth, and three for MIC on agar for a total of 23 QTL.

**Table 1:** Location of QTL identified in this study and the amount of phenotypic variance they explain

Trait	Chr.	Position (cM) (1.5-LOD SI)	Nearest Marker	LOD Score	Genotype	% PVE	Total % PVE	Estimated Effect
Melanin	A	15.6 (11.0, 29.8)	CNA03020	12.5	na	15	49	a 2.9
								d 1.0
	B	15.0 (11.2, 18.8)	CNB03520	13.8	na	16		a 1.9
								d 3.4
	G	3.6 (0.0, 9.6)	CNG01240	9.0	A	10		a −3.9
								d −1.8
	L	0.0 (0.0, 7.2)	CNL03990	11.3	na	13		a 61.1
								d −75.5
	L	9.4 (9.0, 9.8)	CNL06810	10.6	na	12		a −49.0
								d −51.2
								a:a −1.9
	A:B	15.6 (11.0, 29.8)	CNA03020	12.4	AD:AD	14		d:a 6.1
		15.0 (11.2, 18.8)	CNB03520					a:d 5.7
								d:d 16.1
								a:a −126.0
	L:L	0.0 (0.0, 7.2)	CNL03990	8.7	A:A	10		d:a 151.2
		9.4 (9.0, 9.8)	CNL06810					a:d −125.0
								d:d 150.0
Cell Size	A	35.7 (35.0, 37.6)	CNA06130	7.3	AD	8	51	a −0.4
								d −0.7
	A	54.2 (52.4, 58.2)	CNA073100	10.3	na	8		a 0.4
								d 0.7
	A	64.0 (61.0, 64.3)	CNA07990	12.8	na	14		a 0.0
								d 0.1
	D	41.6 (15.6, 41.9)	RUM1	4.5	AD	5		a 0.1
								d 0.4
	F	1.2 (0.0, 8.0)	CNF00290	11.9	na	13		a 0.1
								d 0.2
	I	2.6 (0.0, 12.8)	CNI01350	7.9	na	8		a −0.2
								d 0.3
								a:a −0.2
	A:I	54.2 (52.4, 58.2)	CNA07310	5.5	AD:AD	5.7		d:a −0.6
		2.6 (0.0, 12.8)	CNI01350					a:d −0.2
								d:d 0.3
								a:a −0.2
	A:F	64.0 (61.0, 64.3)	CNA07990	10.8	D:AD	11.9		d:a 0.4
		1.2 (0.0, 8.0)	CNF00290					a:d 0.6
								d:d −0.3
Cell wall size	A	14.6 (3.8, 37.0)	CNA02700	4.3	AD	8	8	a 0.01
								d 0.02
Capsule	A	0.0 (0.0, 33.2)	CNA00050	4.1	AD	5	43	a −0.05
								d 0.04
	B	0.6 (0.0, 3.0)	CNB00360	6.9	na	9		a −0.02
								d 0.07
	D	41.9 (38.6, 41.9)	CND06160	7.9	AD	11		a 0.02
								d 0.10
	H	0.0 (0.0, 8.4)	CNH00030	6.0	na	7		a −0.06
								d 0.05
	L	2.2 (0.0, 10.2)	CNL04620	2.7	AD	3		a 0.02
								d 0.08
								a:a −0.03
	B:D	0.6 (0.0, 3.0)	CNB00360	5.8	AD:AD	7.0		d:a −0.02
		41.9 (38.6, 41.9)	CND06160					a:d 0.02
								d:d 0.3
MIC on agar	A	4.2 (3.0, 6.0)	CNA00290	25.2	AD	36	46	a −1.0
								d 1.0
	E	7.8 (0.0, 26.0)	CNE01630	3.1	AD	3		a −0.2
								d 0.5
	N	15.0 (1.8, 15.0)	CNN02060	3.8	A	4		a 0.3
								d −0.4
MIC in broth	A	4.2 (0.0, 6.0)	CNA00290	9.6	A	14	43	a -1.5
								d 0.3
	A	59.8 (54.0, 64.3)	CNA07470	7.6	AD	11		a −1.2
								d 0.9
	C	9.4 (0, 16.8)	CNC06110	3.2	AD	4		a 0.7
								d 1.1

Positions of QTL (quantitative trait loci) are shown with 1.5–LOD support intervals. Genotype refers to the allele(s) for which the greatest phenotypic values were observed for the given trait, with “na” indicating the marker showing QTL effects in the model for interactive effects. For the estimated effects of each QTL, “a” refers to the additive effect and “d” refers to the dominance effect. For QTL that showed significant interaction these are denoted with a “:”. Chr., chromosome; LOD SI, logarithm of odds support interval; PVE, percent of phenotypic variance explained; MIC, minimal inhibitory concentration.

For simplicity, CDC15 alleles are annotated as A alleles (representing serotype A) and JEC20 alleles as D alleles (representing serotype D). The following subsections are each paired with a figure that displays the significant QTL (as determined by the stepwise command of R/qtl) along with the average values for the given trait for each genotype of the aforementioned QTL. If a QTL was identified at a pseudomarker, rather than a marker, the notation “chromosome@position” was used. If two QTL were found to have a significant interaction effect, both were plotted on the same graph.

### Melanin

**Figure 1:**
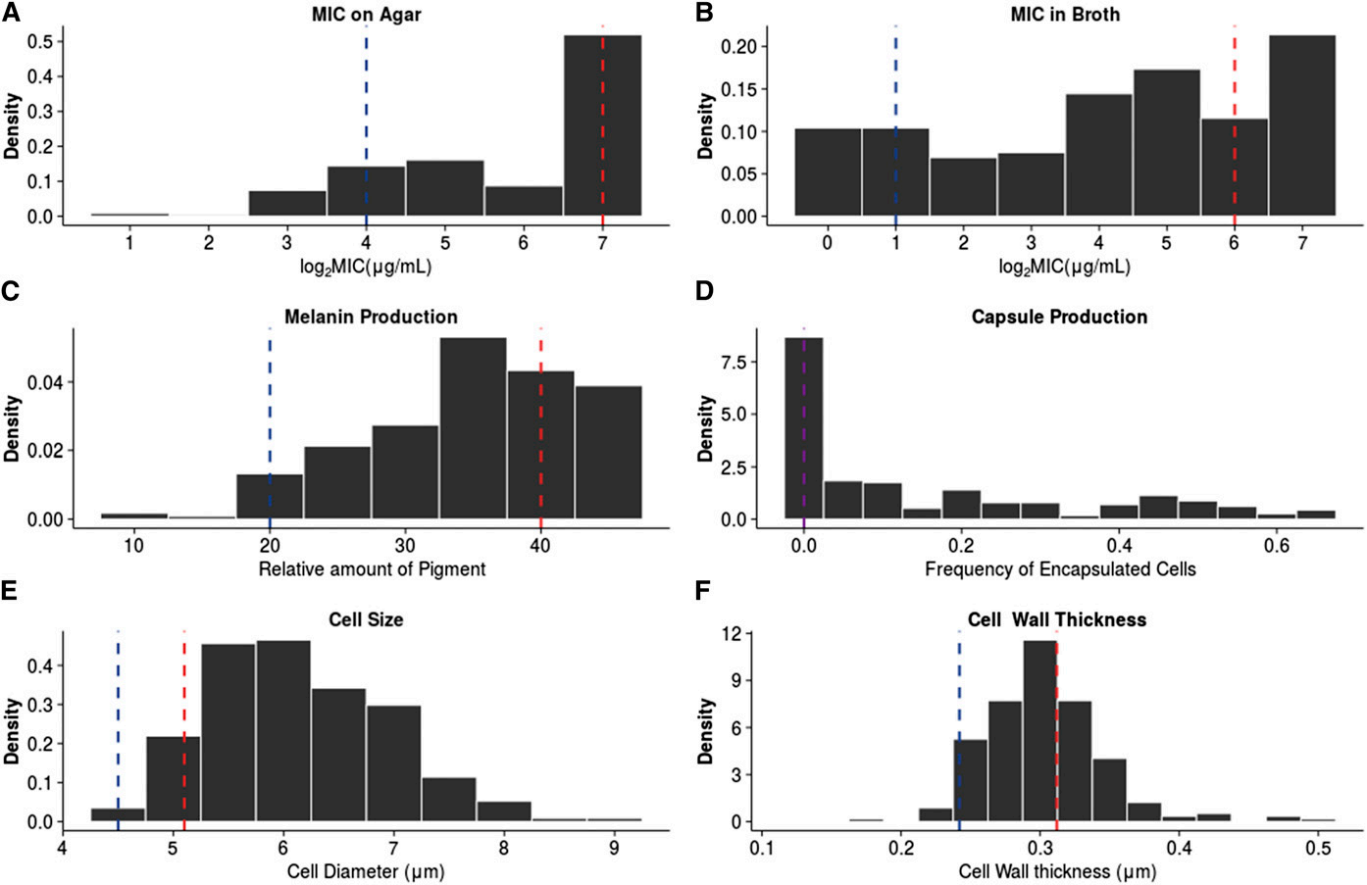
Distribution of virulence traits. Dashed lines represent the means of the parental strains JEC20 (blue) and CDC15 (red) or both (purple). (A) MIC on YEPD agar. (B) MIC in liquid RPMI media. (C) Amount of melanin produced after 3 d. (D) Percent of cells that produce capsule after 2 d in inducing media. (E) Cell diameter (*μ*m). (F) Cell wall thickness (*μ*m). MIC, minimal inhibitory concentration; RPMI, Roswell Park Memorial Institute; YEPD, yeast extract peptone dextrose.

**Figure 2:**
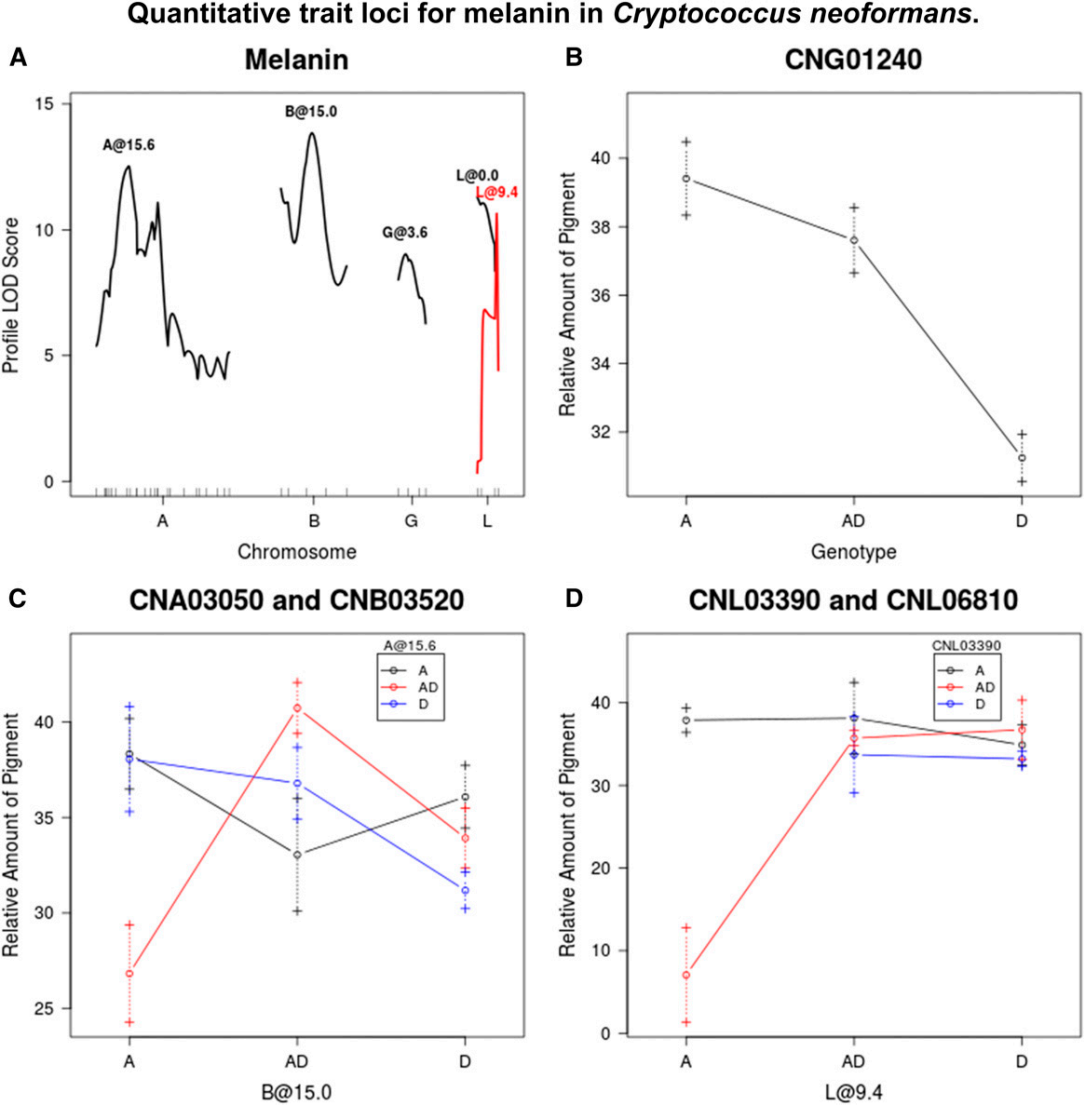
(A) QTL identified for melanin production. (B–D) Average melanin production for given genotypes/genotypic combinations at markers closest to QTL, or at chromosome position if no marker is nearby. Dashed lines represent standard error in the mean. LOD, logarithm of odds; QTL, quantitative trait loci.

The melanin production of JEC20 was significantly lower than that of CDC15 after 7 d (*P* = 1.4 × 10^−6^) (Figure 1). Among the hybrid progeny, melanin values both higher than CDC15 and lower than JEC20 were observed, with five of the 230 progeny showing no melanin production. Five QTL were identified for the production of melanin around markers CNA03050, CNB03520, CNG01240, CNL03390, and CNL06810, which together explain 49% of the phenotypic variance. For the QTL at marker CNG01240, strains which are homozygous for the A allele or heterozygous with both the A and D alleles produce more melanin than strains that are homozygous for the D allele. The QTL at markers CNA03050 and CNB03520 exhibit an interaction effect whereby strains that are heterozygous at both markers produce more melanin than all other allelic combinations. However, strains that are heterozygous at CNA03050, but homozygous A at CNB03520, show the lowest amount of melanin production. QTL by markers CNL03390 and CNL06810 also show an interaction effect, however all allelic combinations display roughly equal amounts of melanin production, except for strains that are heterozygous at CNL03390 and homozygous A at CNL06810. A closer inspection of the data reveals that only one strain has the aforementioned allelic combination, thus this result may be a false positive (Figure 2).

### Cell size

**Figure 3:**
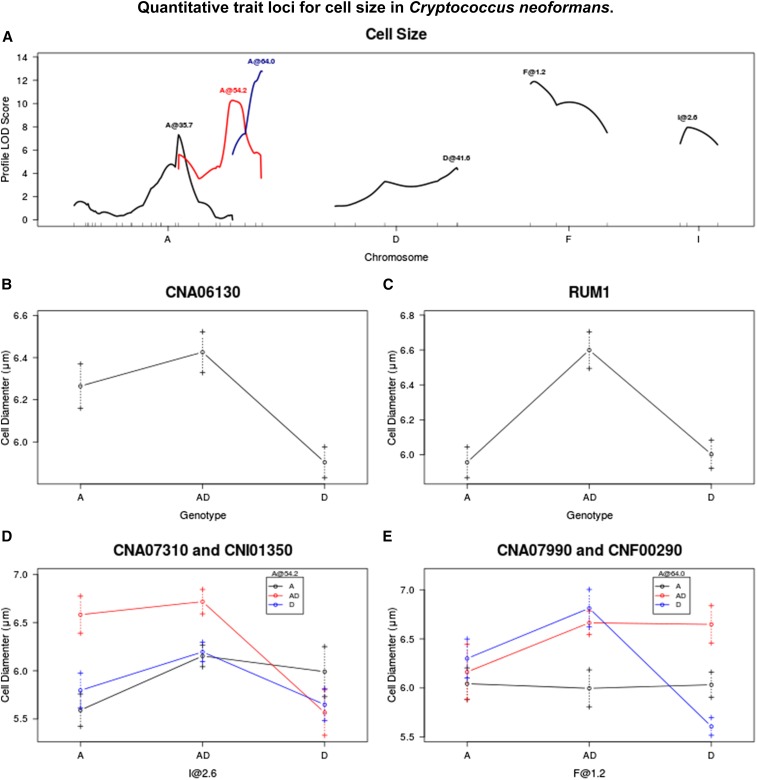
(A) QTL identified for cell size. (B–F) Average cell diameter for given genotypes/genotypic combinations at markers closest to QTL, or at chromosome position if no marker is nearby. Dashed lines represent standard error in the mean. LOD, logarithm of odds; QTL, quantitative trait loci.

Cell diameter differed significantly between JEC20 and CDC15 when they were grown in our specific medium (*P* = 0.001) (see *Materials and Methods*) (Figure 1). Six QTL were identified, around markers CNA06130, CNA07310, CNA07990, RUM1, CNF00290, and CNI01350. The full model explains 51% of phenotypic variance. Strains that are homozygous A or heterozygous at marker CNA06130 are larger than those that are homozygous D. At RUM1, strains that are heterozygous are larger than both homozygous A and homozygous D genotypes. QTL at markers CNA07310 and CNI01350 show an interaction effect, where strains that are heterozygous at CNA07310 and heterozygous or homozygous A at marker CNI01350 are larger than strains of other genotypic combinations at these two loci. QTL at markers CNA07990 and CNF00290 also show an interaction effect. Strains that are heterozygous at both loci, heterozygous at CNF00290, and homozygous D at CNA07990, or heterozygous at CNA07990 and homozygous D at CNF00290, have larger cells than those with other genotypes. Strains that are homozygous D at both marker loci have the smallest cell sizes (Figure 3).

### Cell wall thickness

**Figure 4:**
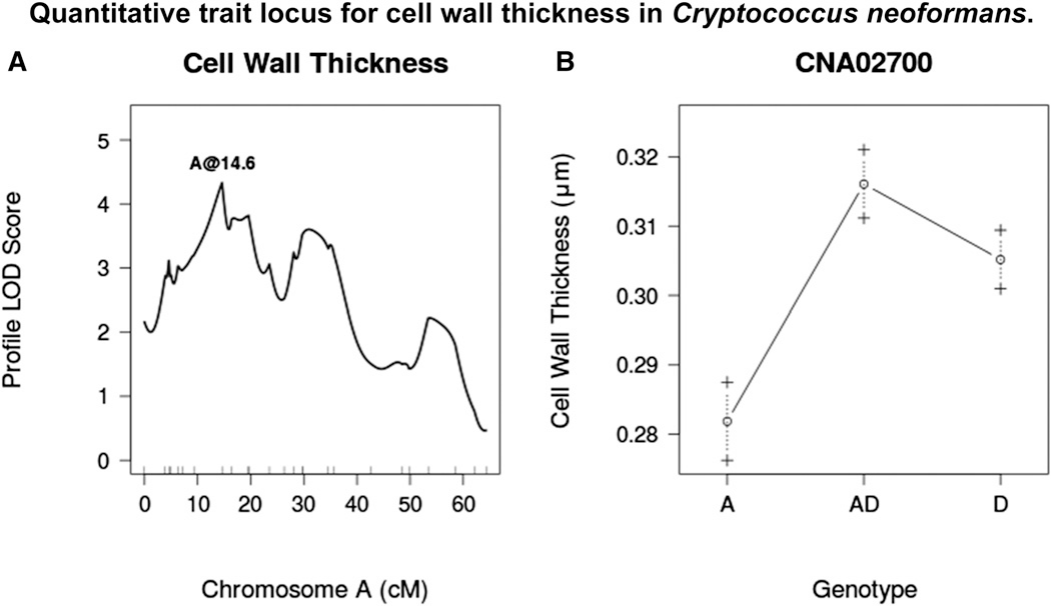
(A) LOD score profile for a QTL associated with cell wall thickness. (B) Average cell wall thickness for given genotypes at marker CNA02700. Dashed lines represent standard error in the mean. LOD, logarithm of odds; QTL, quantitative trait loci.

Cell wall thickness was found to differ significantly between JEC20 and CDC15 (*P* = $4.8\times 10^{-6}$) (Figure 1). Under noninducing conditions, cell wall thickness has been reported as ∼0.05 μm (Feldmesser *et al.* 2001). We observed a range of values from 0.17 μm to 0.49 μm across all strains, indicating a 4–10-fold increase in cell wall thickness over noninducing conditions. A single QTL was identified on chromosome A at marker CNA02700 that explains 8% of the phenotypic variance. Strains that are heterozygous or homozygous D at this locus have thicker cell walls than those that are homozygous A (Figure 4).

### Capsule production

**Figure 5:**
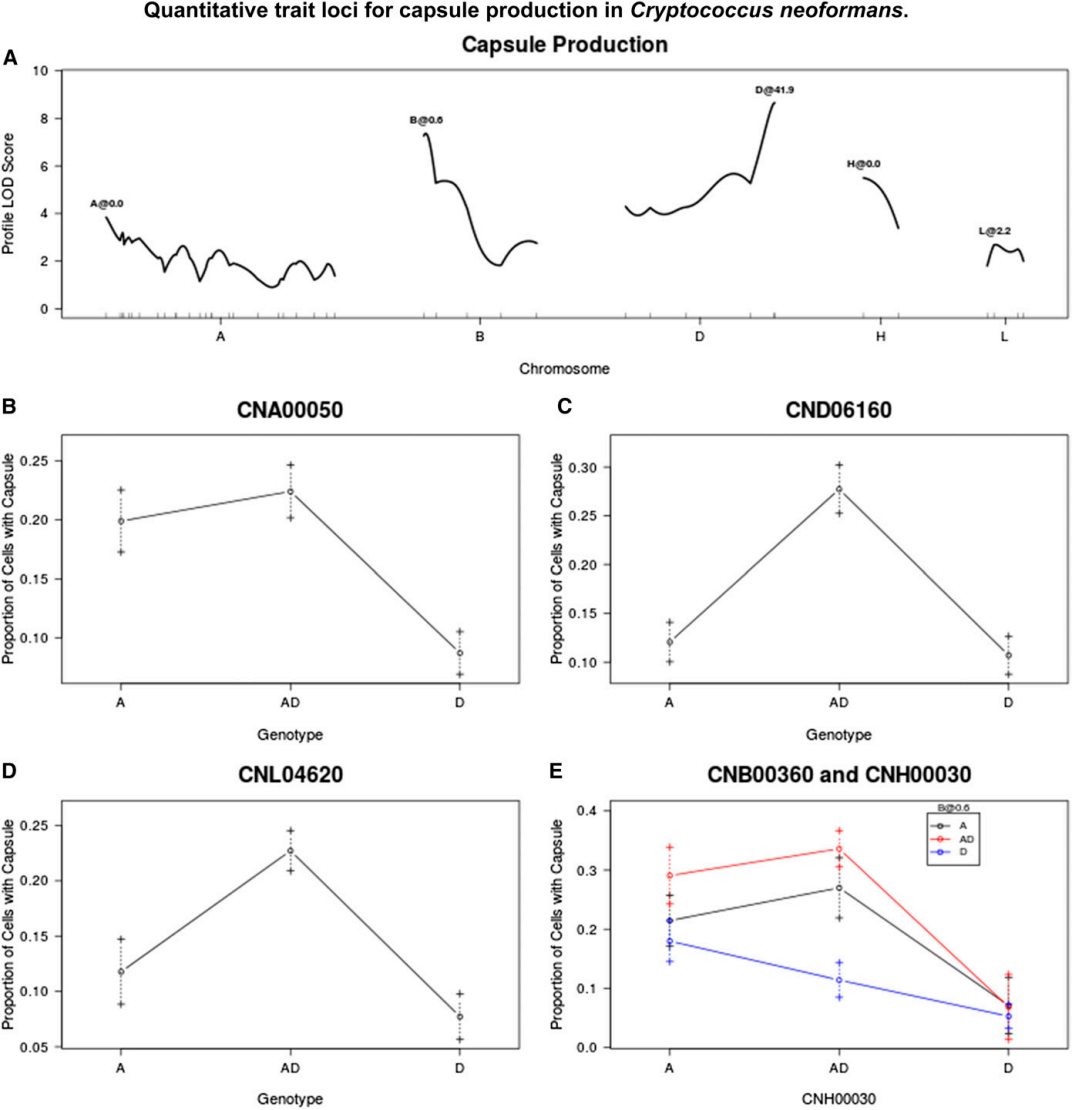
(A) QTL identified for capsule production. (B–E) Average proportion of cells producing capsule for given genotypes/genotypic combinations at markers closest to QTL, or at chromosome position if no marker is nearby. Dashed lines represent standard error in the mean. LOD, logarithm of odds; QTL, quantitative trait loci.

Under noninducing conditions, no capsule was produced by either JEC20 or CDC15. Under inducing conditions, there was still no capsule production after 2 d for the two parental strains; however, after 7 d, capsule production was observed for some CDC15 cells, but little to no production was noted for JEC20. It was not possible to measure this small amount of capsule with the technique applied here. Among the progeny, capsule production was very heterogeneous. Many strains produced some cells that had large quantities of capsule after only 2 d, while other cells showed no capsule. When capsule area was used as a phenotypic trait, measured only for cells that produced at least some measurable amount of capsule and controlled for cell size, no QTL were identified. When the frequency of capsule production, that is the proportion of cells that produced at least some measurable amount of capsule per strain, was analyzed as a quantitative trait, five QTL were identified. These were around markers CNA00050, CNB00360, CND06160 CNH00030, and CNL04620, which together explain 43% of the phenotypic variance. At marker CNA00050, strains that are homozygous A or heterozygous produce capsule at a higher frequency than those that are homozygous D. At markers CND06160 and CNH0030, strains that are heterozygous produce capsule more frequently than those that are homozygous for either parental allele. QTL by markers CNB00360 and CNH0030 show an interaction effect where strains that are heterozygous at both markers, homozygous A at both markers, or homozygous A at one marker and heterozygous at the other marker produce capsule more frequently than strains that are homozygous D at both markers, or heterozygous at CNH00030 and homozygous D at CNB00360 (Figure 5).

### Fluconazole resistance

**Figure 6:**
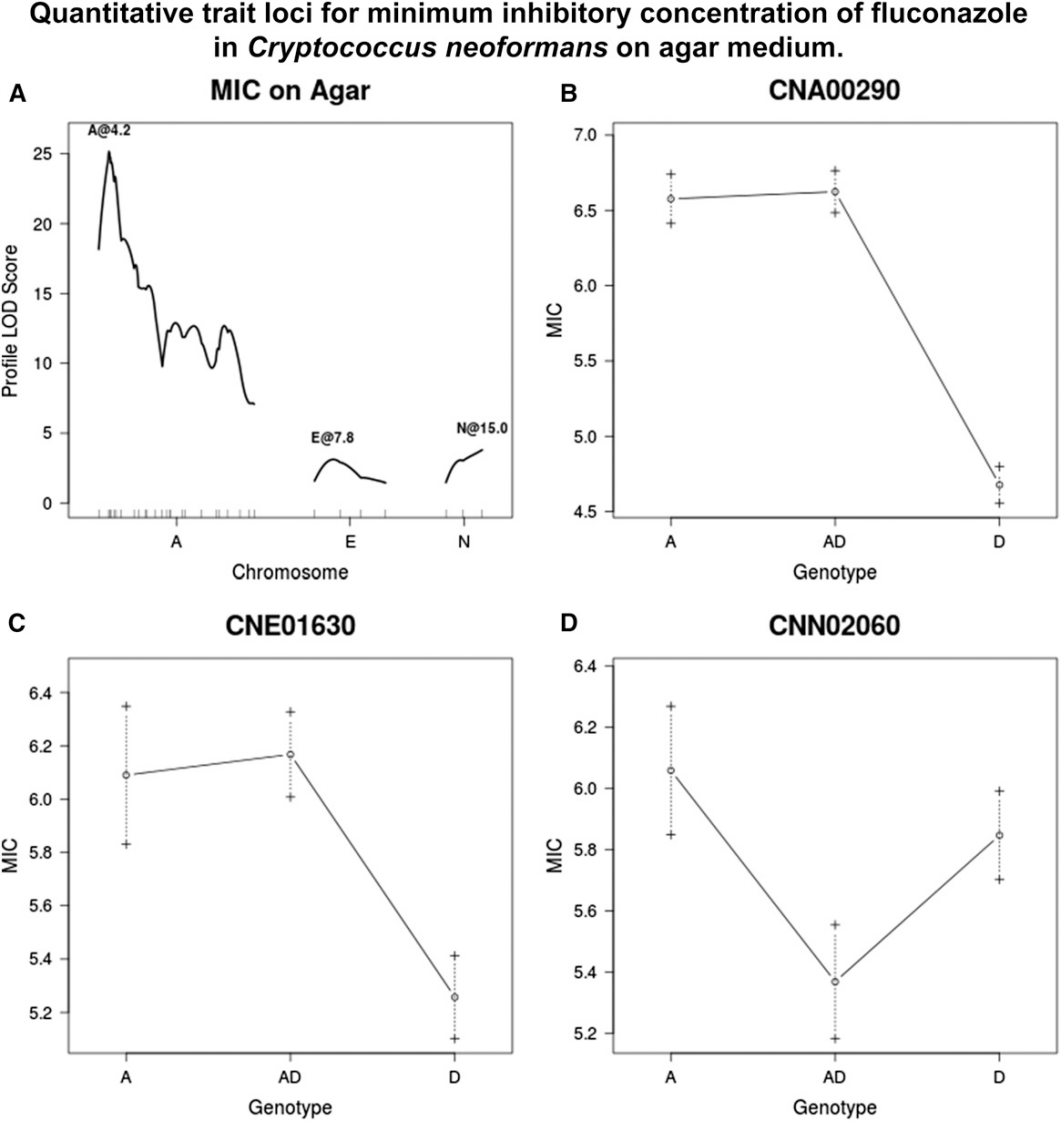
(A) QTL identified for fluconazole resistance on agar. (B–D) Average MIC when grown on agar for given genotypes/genotypic combinations at markers closest to QTL, or at chromosome position if no marker is nearby. Dashed lines represent standard error in the mean. LOD, logarithm of odds; MIC, minimal inhibitory concentration; QTL, quantitative trait loci.

JEC20 has an MIC of 16 μg/ml when measured on solid agar plates and an MIC of 4 μg/ml in broth for fluconazole. CDC15 has an MIC of 128 μg/ml on agar and an MIC of 32 μg/ml in broth for fluconazole. Overall, the progeny had average MIC values lower than the parental mean. However, a number of strains had MICs of 128 μg/ml in broth (Figure 1). Three QTL were identified for MIC on agar at markers CNA00290, CNE01630, and CNN02060. The QTL at CNA00290 has the largest effect by far, explaining 36% of the phenotypic variance on its own. By contrast, the model as a whole explains 46% of the phenotypic variance. Progeny that are homozygous A or heterozygous at markers CNA00290 and CNE01630 have a higher MIC than those that are homozygous D at this marker. Strains that are homozygous A at CNN02060 have higher MICs than those that are heterozygous (Figure 6).

**Figure 7:**
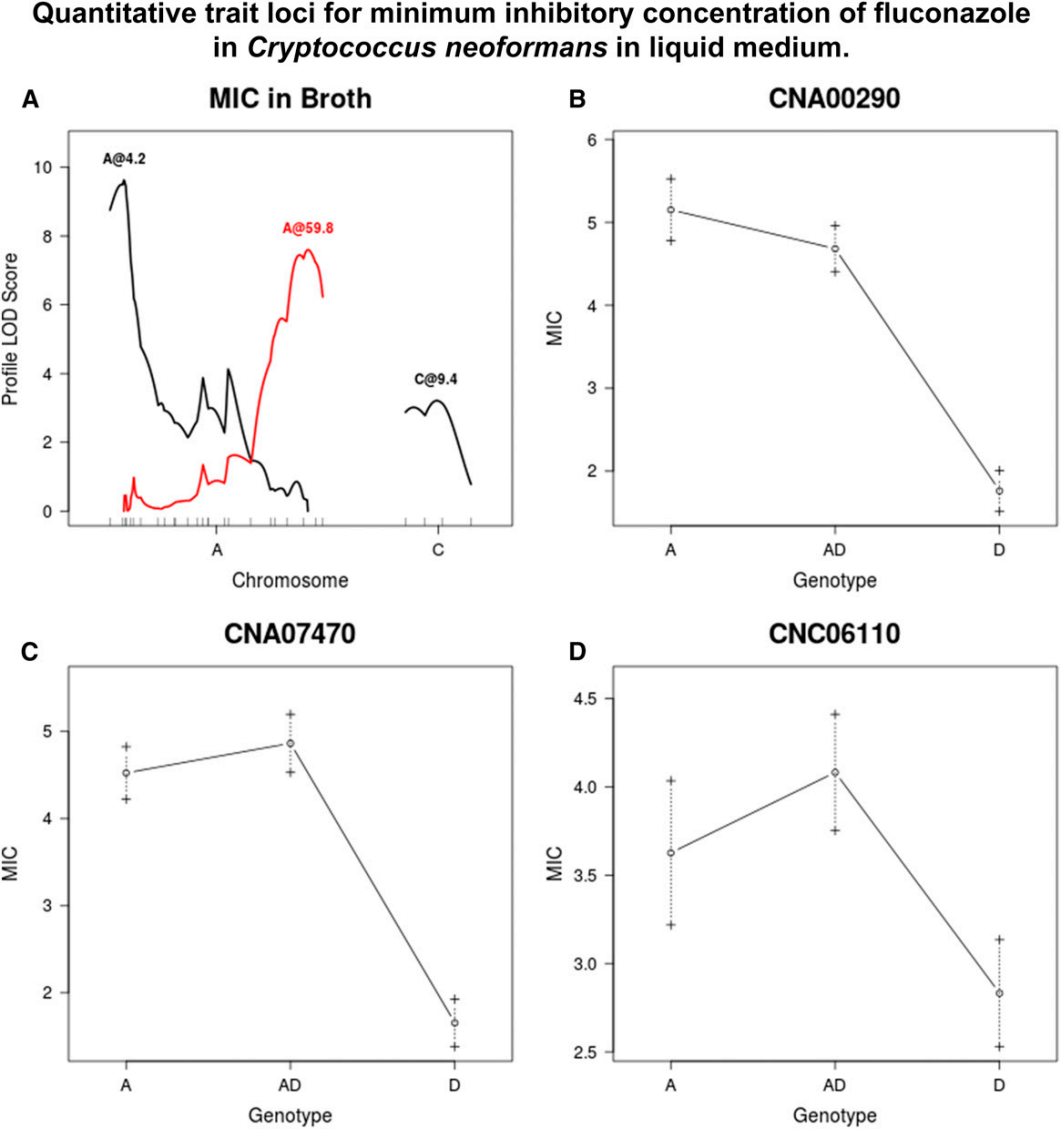
(A) QTL identified for fluconazole resistance in broth. (B–D) Average MIC when grown in broth for given genotypes/genotypic combinations at markers closest to QTL, or at chromosome position if no marker is nearby. Dashed lines represent standard error in the mean. LOD, logarithm of odds; MIC, minimal inhibitory concentration; QTL, quantitative trait loci.

The results from broth also showed three QTL; again there was one at CNA00290, similar to what was found on solid medium. The other two were different from those identified based on data from agar plates, located by markers CNA07470 and CNC06110. The full model explains 44% of phenotypic variance. All markers show a similar trend, whereby strains that are homozygous A or heterozygous for a given marker have a higher MIC than those that are homozygous D (Figure 7).

**Figure 8:**
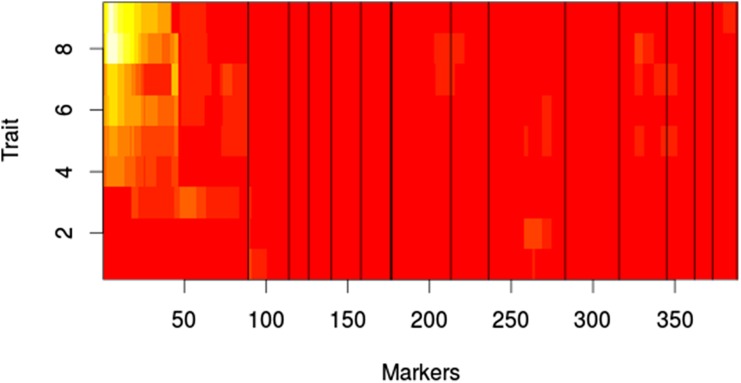
MQM results for growth with fluconazole on agar. White indicates high LOD score. Red indicates low LOD score. X-axis shows log ${}_{2}\mu$M of fluconazole. Markers represent pseudomarkers placed throughout the linkage map. Chromosomes are indicated by black vertical lines. LOD, logarithm of odds; MQM, multiple QTL mapping; QTL, quantitative trait loci.

**Figure 9:**
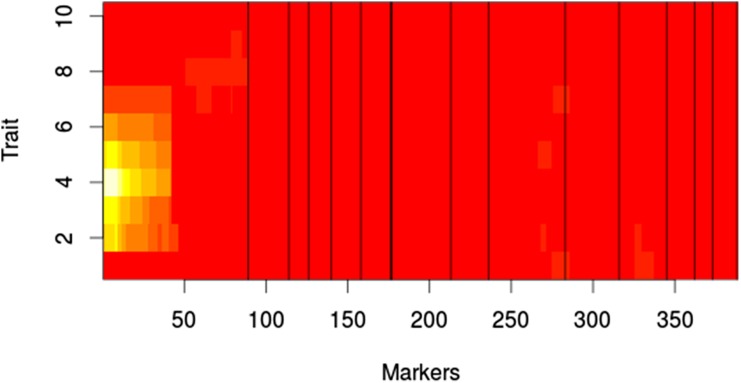
MQM results for growth with fluconazole in liquid RPMI. White indicates high LOD score. Red indicates low LOD score. X-axis shows log ${}_{2}\mu$M of fluconazole. Markers represent pseudomarkers placed throughout the linkage map. Chromosomes are indicated by black vertical lines. LOD, logarithm of odds; MQM, multiple QTL mapping; QTL, quantitative trait loci; RPMI, Roswell Park Memorial Institute.

The results from the MQM analysis were consistent with the results from the MIM evaluation of MIC. For growth on YEPD agar with fluconazole, marker CNA00290 shows the highest LOD score at concentrations above 1.0 μg/ml, with the rest of the markers contributing very little (Figure 8). At 1.0 μg/ml of fluconazole, marker CNA05300 shows the highest LOD score; however, at 0.5 μg/ml, CNA00290 once again shows the highest LOD score. For growth on RPMI with fluconazole, marker CNA00290 has the highest LOD scores for concentrations of 0.5 μg/ml to 16.0 μg/ml, with a peak LOD score at 2.0 μg/ml (Figure 9). No QTL were observed at 0 μg/ml, 64.0 μg/ml, and 128.0 μg/ml. In agreement with the MIM results in broth, at 32.0 μg/ml marker CNA07470 has the highest LOD score.

## Discussion

In this study we have identified 23 QTL that contribute to the differential manifestation of several phenotypes between a laboratory strain of *C. deneoformans* (JEC20) and a clinical, drug resistant isolate of *C. neoformans* (CDC15). Among these, we have identified six QTL involved in resistance to the azole drug fluconazole. Interestingly, all of the six traits examined in this study possessed QTL on chromosome A. Chromosome A is the largest chromosome, containing approximately 13% of the genes in genome. Given this and the total number of QTL, the observed distribution of QTL among the chromosomes is not unexpected (*P* = 0.17, chi square distribution).

### Linkage map

Overall, the linkage map generated from the mapping population was larger than that previously reported by Sun and Xu (322.5 cM *vs.* 196.8 cM), which used identical parental strains (Sun and Xu 2007). On a chromosome by chromosome basis, every chromosome in the linkage map generated here showed greater map distance than that in the earlier paper, with the exceptions of chromosomes H and K. Because many of the markers used for genotyping were identical between the two studies, minor differences in the markers cannot account for the discrepancy in recombination frequencies and linkage map size. However, the mapping population collected for this study was obtained through micromanipulation and we waited for an extended period of time to allow all viable progenies to germinate (Vogan *et al.* 2013). In contrast, those collected in the earlier study by Sun and Xu were obtained through random spore spread-plating, where fast germinating and fast growing progeny were likely picked for analyses. The inclusion of slow growing progeny in the mapping population likely increased the number of recombination events and map length, as observed in this study.

### QTL mapping

The primary goal of this work was to identify the regions of the genome where a difference in alleles contributes to major phenotypic differences between strains of two divergent lineages in the pathogenic *Cryptococcus* species complex. We would like to note that interpreting the observed differences can be complicated by the total number of chromosomes among the hybrid progeny. Ideally, one would conduct this study with only haploid progeny or only diploid progeny from the hybrid cross. However, out of the 230 progeny examined here, only six strains showed no heterozygosity (indicative of being fully haploid) and only 14 were heterozygous for at least one marker per chromosome (indicative of being fully diploid). For the remaining progeny, the copy numbers of genes and chromosomes likely vary between one and two copies per cell.

In the QTL mapping analyses, we assumed that all hybrid progeny were diploid. However, a significant proportion of the progeny may be aneuploid and it has been demonstrated that disomy of specific chromosomes in *C. neoformans* can lead to fluconazole resistance and increased virulence in mice (Sionov *et al.* 2010, 2009). Additionally, it has been demonstrated in *Saccharomyces cerevisiae* that the beneficial effects of chromosomal duplication are a result of a few individual loci on the specific chromosome rather than a chromosome-wide response (Sunshine *et al.* 2015). It is therefore possible that the QTL identified here as associated with higher levels of fluconazole resistance or virulence-associated traits may be a result of variations in the copy numbers of genes and chromosomes. In our analyses, all homozygous genotypes were coded as AA or DD. In actuality, a portion of the homozygous loci are likely A– or D– due to chromosome loss (Vogan *et al.* 2013). As such, if there is a benefit to having two copies of a given locus regardless of the specific allele, then the copy number effect or heterosis effect may be masked. This is because all heterozygous loci should be present as two copies, whereas the pool of homozygous genotypes will be represented by individuals with either one or two copies of the gene.

Following this line of reasoning, we analyzed all of the QTL for evidence of the effect of copy number on the traits. To do this, genotypes which were determined to be A or D were subdivided into AA, A–, or A?, and DD, D–, or D?, based on the genotype information at surrounding markers on the same chromosome. For progeny with either the A or D allele at the locus of interest, but heterozygous (H) at at least one other locus on the same chromosome, they were categorized as AA or DD. If at least one other locus on the same chromosome was the opposite genotype of the locus of interest, but no heterozygous loci were present on that chromosome, they were categorized as A– or D–. This assumes that we have genotyped a sufficient number of markers on each chromosome as to observe heterozygosity were it present. If the entire chromosome was homozygous for the given allele, they were categorized as A? or D?. See Vogan *et al.* (2013) for a detailed explanation as to why the copy number of fully homozygous chromosomes cannot be determined (Vogan *et al.* 2013). If the number of alleles at a given locus was the sole reason for larger values of a given trait, then H, AA, and DD categories would be significantly different than A– or D–, but would not differ significantly from each other. However, this was never observed in our data set. For two cases (cell size at RUM1 and capsule production at CND06160) the results suggest that there may be a combined effect of copy number and heterosis. Specifically, for these two traits, progeny that are H have significantly higher values than those that are A– or D–, indicative of heterosis. However, there are no differences between the AA and DD genotypes or between them and any other genotypes (Table S1).

### Melanin production

The production of melanin is also an important virulence factor in other human pathogenic fungi, such as *Paracoccidioides brasiliensis* and *Histoplasma capsulatum* (Taborda *et al.* 2008), in plant pathogens such as *Magnaportha grisea* (Chumley *et al.* 1990), and in insect pathogens like *Metarhizium anisopliae* (Fang *et al.* 2010). The biosynthetic pathways responsible for the production of melanin have been identified in many of these fungi; however, little is known about the regulatory networks that influence the expression of these genes (Yu and Keller 2005). In *Cryptococcus*, the transcription factors PKA1 (D’Souza *et al.* 2001), PKA2 (Hicks *et al.* 2004), GPA1 (Pukkila-Worley *et al.* 2005), and MAC1 (CNG02270) (Lin *et al.* 2006) are required for the production of melanin, though the effects of knockouts with these genes show broad scale impacts to multiple virulence factors, rather than specificity to melanin.

Previous studies have shown that the transcription factor PKA1 regulates virulence factors in *C. neoformans*, but not in *C. deneoformans* (Hicks *et al.* 2004). This gene lies close to locus CNA03840, the region of the QTL identified on chromosome A for melanin production (Figure 2). Currently, it is not known how PKA1 acts to induce an effect on melanin production (Idnurm *et al.* 2005). The results here indicate that this QTL interacts with another QTL, located on chromosome B. Identifying the gene could help reveal the genetic basis for differences in melanin production between the two species. At present, there is no known gene at this region on chromosome B that may interact with PKA1.

One of the QTL identified for melanin production is located near marker CNG01240. This marker lies at/close to the LAC1 gene, which codes for laccase in *C. neoformans* (Williamson 1994). Laccase is responsible for the key enzymatic step in the conversion of precursors, such as L-DOPA, into melanin and is necessary for virulence (Liu *et al.* 1999; Salas *et al.* 1996). There are numerous ($\frac{61}{265}$) amino acid substitutions between the sequences for laccase in the reference genomes of H99 (*C. neoformans*) and JEC21 (*C. deneoformans*), but relatively little within-species sequence divergence (Ito-Kuwa *et al.* 2008). However, no studies have shown whether or not these amino acid changes affect melanin production differences between these two lineages. A detailed promoter analysis has been conducted on *C. deneoformans* and determined that multiple transcription factors control melanin expression (Zhu and Williamson 2004), but a parallel experiment has not been conducted in *C. neoformans*. A comparative analysis using *in silico* methods is not viable as the ORF CNAG_07734 is present immediately upstream of LAC1 in *C. neoformans*, but absent in *C. deneoformans*. A follow-up experiment to replace the JEC20 allele with the CDC15 allele in JEC20 or vice versa at the LAC1 locus could help understand whether these amino acid differences contribute to melanin expression differences between the two strains.

Aside from LAC1, there is another candidate gene [MAC1 (locus CNG02270)] at this QTL region. The influence of MAC1 on melanin production was discovered through previous QTL mapping in *C. deneoformans* by Lin *et al.* (2006). The cross in that study was between two inbred strains of *C. deneoformans* that were specifically selected for their extreme phenotypes regarding the length of hyphae produced during self-mating (Lin *et al.* 2006). This gene is at the locus CNG02260, which is tightly linked to LAC1 in our linkage map. As such, it cannot be excluded as a candidate for the QTL on chromosome G. Indeed, it is possible that both genes could have contributed to the observed QTL effects in this region.

### Cell size and cell wall thickness

Measurements for cell size and cell wall thickness were conducted under nutrient limiting conditions. Previously, Feldmesser *et al.* observed cell wall thickening and an increase in cell size during the course of pulmonary infection in a mouse model (Feldmesser *et al.* 2001) with *C. deneoformans*. After 48 hr of infection, they reported the ratio of cell wall thickness to cell diameter as 0.061 ± 0.026 as compared to 0.48 ± 0.020 5 min postinfection. In agreement with this work, we found that after 48 hr of growth in minimal medium, this ratio was 0.10 ± 0.032 for JEC20 and 0.14 ± 0.021 for CDC15. This suggests that starvation induces a similar physiological response as the host environment in *Cryptococcus*.

The QTL identified for cell wall thickness displays an unexpected pattern of association between genotype and cell wall thickness. Despite the fact that cells from CDC15 have thicker cell walls than JEC20 under the inducing condition, in the hybrid progeny, strains that are homozygous D or heterozygous have thicker cell walls than homozygous A progeny (Figure 4). This suggests that the D allele at this locus promotes cell wall thickening in a hybrid background, but may have little or no effect in the JEC20 background. Alternatively, there may be a second interacting locus that is necessary for increased cell wall thickening in CDC15, but not in JEC20. However, we were unable to identify such a locus using the present data.

Interestingly, three of the five QTL associated with cell size were located on chromosome A (Figure 3). Our result is consistent with previous observations about the importance of chromosome A to cell size. For example, Sionov *et al.* (2010) reported that a clonal descendant of strain H99 but with two copies of chromosome A exhibited increased cell size (Sionov *et al.* 2010). Research on the model yeast *Saccharomyces cerevisiae* has revealed that factors controlling cell cycle can effect cell size in a ploidy-dependent manner, by lengthening the time a cell spends in the G1 phase of the cell cycle (Talia *et al.* 2007). Thus, the genes underlying these three QTL may code for cyclins or cyclin-dependent products, though none have been identified as such in the annotated genome of JEC21.

Given the large amount of correlation between cell size and the other traits (Table S5), the QTL identified by marker CNA07470 for MIC in broth, and by marker CND06160 for capsule production, may be a result of pleiotropic effects with cell size, rather than specific effects of those traits.

### Capsule production

As neither JEC20 nor CDC15 showed any capsule production after 2 d in the inducing medium, it was expected that any QTL for capsule production would be associated with heterozygosity and/or aneuploidy. However, this was not the case for the QTL at marker CNA00050, one of the five QTL identified associated with the frequency of capsule production. The reasons for this phenomenon are not known.

As with melanin production, the transcription factors PKA1, PKA2, and GPA1 have been shown to influence capsule production and virulence in general, along with the regulatory subunit of PKA1, PKR1 (Hicks *et al.* 2004). According to our QTL mapping, none of these genes appear to affect capsule production in the hybrids. Additionally, there are upwards of 35 other genes, which have been implicated in capsule biosynthesis (O’Meara and Alspaugh 2012). This large number of genes, combined with the large support intervals around the QTL identified here, results in numerous candidate genes for the QTL identified on chromosomes A and L. The QTL identified on chromosomes B, D, and H have only a single candidate gene. The QTL on chromosome D is close to the MAT locus, which contains the gene CAP1 (locus CND05940). CAP1 has a high similarity to the gene CAP10, which is important for capsule biosynthesis, but its exact function has not yet been elucidated (Lengeler *et al.* 2002). The genes CAS3 (locus CNB01440) and PBX2 (locus CNH02100) lie within the regions identified for the QTL on chromosomes B and H, respectively. These two QTL also exhibit an interactive effect with each other (Figure 5). To date, no studies have provided direct evidence that these genes, or their products, interact with each other. However, both genes are known to be involved in the biosynthesis of glucuronoxylomannan (GXM), one of the two main sugar components of the capsule (Liu *et al.* 2007). GXM shows numerous compositional differences between *C. neoformans* and *C. deneoformans* and may be responsible for the difference in serology between the species in general (McFadden *et al.* 2006). As yet, no gene has been identified that controls serotype in *Cryptococcus*. These results suggest that CAS3 and PBX2 may be prime candidates for differences in capsule production between the two strains, but it is important to note that these genes were identified based on the production of capsule and not on the structure of it.

A minor complication to the traits measured for cell size, cell wall thickness, and frequency of capsule production is that we assumed that all the measured cells for a given progeny have exactly the same genotype. Previously, we demonstrated that a loss of heterozygosity could happen among separate colonies isolated from the same strain in this mapping population (Vogan *et al.* 2013). For most of the traits analyzed here, we can safely assume that the phenotypes represent the major genotype of a given colony, but individual cell measurements mean that some cells may have genotypes different from the majority of the cells. If these different genotypes cause large shifts in the phenotype of that cell, then a simple average may not represent the phenotype appropriately. However, the average coefficient of variation for all of the traits that required single cell measurements was 0.33, and only 5% of measurements had coefficients of variation above 0.5, suggesting that this might not be a significant confounding factor for these traits.

### Fluconazole resistance

Ongoing work in our lab has revealed that CDC15 contains a well-known point mutation that confers resistance to fluconazole, namely Y145F in ERG11 (the gene that codes for the target of fluconazole) (Sionov *et al.* 2012). This gene lies adjacent to marker CNA00290, which is the location of the very strong QTL identified in all of the analyses for fluconazole resistance. Because of the strong effect of this mutation, it was unexpected to find that for the MQM analysis of MIC in liquid media, the QTL by marker CNA07470 was the only QTL identified at high drug concentrations. ERG2 (locus CNA08290), a gene involved in the ergosterol biosynthetic pathway, is located in this region. Its position in the pathway is downstream from ERG11, and CDC15 possess no known mutations in this gene related to azole resistance. ERG9 (locus CNC05660) and ERG25 (locus CNC02410) code for enzymes upstream and downstream of ERG11, respectively. They reside in the region of the QTL identified for MIC in broth on chromosome C. Both have shown increased transcription levels in response to azole drugs in *S. cerevisiae* (Bammert and Fostel 2000). None of the other QTL identified for fluconazole resistance have any candidate genes with known roles in azole resistance (Lupetti *et al.* 2002).

Previous work has determined that disomy at chromosome A results in higher MICs of laboratory strains, and may be a result of increased levels of the ERG11 protein (Sionov *et al.* 2010). Additionally, Sionov *et al.* (2010) presented convincing evidence that disomy at chromosomes 4, 10, and 14 of strain H99 also contributed to azole resistance. These chromosomes correspond to Chromosomes L, J, and H as annotated here. However, none of the other four QTL identified for MIC on agar and in broth are located on these chromosomes, suggesting that there are additional loci contributing to fluconazole resistance in *C. neoformans*.

### Conclusions

Here, we have identified 23 QTL that are associated with differences in the expression of virulence factors and in susceptibility to the antifungal drug fluconazole. Heterozygous advantages were found for several of the traits investigated here. One of the major evolutionary questions about *Cryptococcus* is whether the large difference in virulence observed between *C. neoformans* and *C. deneoformans* is the result of differential environmental adaptation that coincidentally provides a means to cause infections in humans, or alternatively the result of direct selection for virulence in *C. neoformans*. Identifying the genes that control the differential expression of virulence factors between these species will ultimately allow this question to be answered. Our results represent an essential step from which to dissect the genetic bases of major phenotypic differences between two strains representing the two divergent lineages of the pathogenic *Cryptococcus* species complex. Further analyses of the candidate genes and gene regions underlying these QTL should help elucidate the genetic mechanisms that allow this normally saprophitic organism to cause deadly diseases in humans.

## Supplementary Material

Supplemental Material
