# Supplementary material for: Identification of QTLs Associated with Virulence Related Traits and Drug Resistance in Cryptococcus neoformans
Source: G3 (Bethesda). 2016 Jun 30;6(9):2745–59. doi: 10.1534/g3.116.029595 (PMC5015932; doi:10.1534/g3.116.029595)
Supplement: Supplemental Material [file supp_6_9_2745__index.html]

Identification of QTLs Associated with Virulence Related Traits and Drug Resistance in Cryptococcus neoformans — Supplemental Material 

# Identification of QTLs Associated with Virulence Related Traits and Drug Resistance in *Cryptococcus neoformans*

## Supplemental Material for Vogan *et al.*, 2016

**Files in this Data Supplement:**

- Table S2 - Average phenotype ± standard deviation for given genotypic class. (.pdf, 91 KB)
- Table S1 - A list of all markers and primers used in this study as well as the restriction enzymes used to distinguish between the parental genotypes. (.xlsx, 22 KB)
- Table S3 - Table used for QTL analysis. (.xlsx, 94 KB)
- Table S4 - Table of detailed output regarding the QTL detection. (.xlsx, 11 KB)
- Table S5 - Detailed results from the correlation analysis. (.xlsx, 5 KB)
