# Supplementary material for: Identification of QTLs Associated with Virulence Related Traits and Drug Resistance in Cryptococcus neoformans
Source: G3 (Bethesda). 2016 Jun 30;6(9):2745–59. doi: 10.1534/g3.116.029595 (PMC5015932; doi:10.1534/g3.116.029595)
Supplement: Supplemental Material [file supp_g3.116.029595_TableS2.pdf]

Table S2: Average phenotype ± standard deviation for given genotypic class. The marker closest to the QTL of interest, as indicated by the position (pos) and Chromosome (Chr), was used for the anlysis.

Trait	**Chr**	**Pos (cM)**	**Marker**	**AA**	**A-**	**A?**	**DD**	**D-**	**D?**	**H**
Melanin	A	15.6	CNA03050	38.21 ± 6.66^{ab}	35.83 ± 7.19^{ab}	36.70 ± 7.27^{ab}	39.08 ± 4.03^{ab}	33.73 ± 5.55^{ab}	31.18 ± 10.14^{b}	36.16 ± 8.89^{a}
	B	15.0	CNB03520	36.56 ± 10.35^{ab}	31.27 ± 9.19^{b}	39.20 ± 5.91^{a}	30.55 ± 11.96^{ab}	32.72 ± 7.07^{ab}	33.03 ± 7.91^{b}	38.40 ± 7.14^{a}
	G	3.6	CNG01240	43.51^{ab}	39.82 ± 4.28^{a}	39.40 ± 5.69^{a}	26.39 ± 8.91^{ab}	33.45 ± 2.80^{ab}	30.88 ± 8.61^{b}	38.03 ± 6.40^{a}
	L	0.0	CNL03990	38.32 ± 5.56^{a}	34.22 ± 7.71^{a}	38.05 ± 5.25^{a}	32.84 ± 7.14^{a}	27.43^{a}	33.23 ± 7.39^{a}	35.03 ± 9.69^{a}
	L	9.4	CNL06810	4.98^{a}	32.14^{ab}	38.05 ± 5.25^{b}	34.38 ± 5.80^{b}	34.87 ± 7.64^{b}	33.23 ± 7.39^{b}	35.29 ± 9.10^{b}
Cell Size	A	35.7	CNA06130	6.63 ± 0.78^{ac}	6.22 ± 0.86^{ab}	6.10 ± 0.54^{ab}	6.42 ± 0.88^{ab}	5.77 ± 0.67^{b}	5.87 ± 0.76^{bc}	6.44 ± 0.81^{a}
	A	54.2	CNA07310	6.39 ± 0.87^{ab}	5.85 ± 0.58^{b}	6.10 ± 0.54^{ab}	6.37 ± 0.83^{ab}	6.10 ± 0.94^{ab}	5.87 ± 0.76^{b}	6.48 ± 0.80^{a}
	A	64.0	CNA07990	6.59 ± 1.00^{ab}	5.91 ± 0.68^{b}	6.10 ± 0.54^{ab}	5.85 ± 0.77^{b}	5.99 ± 0.87^{b}	5.87 ± 0.76^{b}	6.60 ± 0.72^{a}
	D	41.6	RUM1	5.56 ± 0.68^{ab}	5.96 ± 0.75^{b}	5.94 ± 0.67^{b}	6.52 ± 0.82^{ab}	5.85 ± 0.70^{b}	5.99 ± 0.67^{b}	6.57 ± 0.88^{a}
	F	1.2	CNF00290	6.52 ± 0.33^{ab}	6.25 ± 0.77^{ab}	6.08 ± 0.59^{ab}	6.08 ± 0.76^{ab}	5.94 ± 0.69^{b}	5.83 ± 0.78^{b}	6.56 ± 0.81^{a}
	I	2.6	CNI01350	5.91 ± 0.62^{ab}	5.42 ± 0.55^{b}	6.15 ± 0.69^{ab}	5.79 ± 1.28^{ab}	6.03 ± 0.39^{ab}	5.72 ± 0.62^{b}	6.32 ± 0.82^{a}
Cell Wall Thickness	A	14.6	CNA02700	0.05 ± 0.00^{abc}	0.05 ± 0.01^{b}	0.05 ± 0.01^{abc}	0.04 ± 0.01^{ab}	0.05 ± 0.01^{ac}	0.06 ± 0.01^{c}	0.05 ± 0.01^{ac}
Capsule	A	0.0	CNA00050	0.27 ± 0.28^{ab}	0.19 ± 0.21^{ab}	0.22 ± 0.22^{ab}	0.12 ± 0.19^{ab}	0.07 ± 0.14^{b}	0.09 ± 0.16^{b}	0.22 ± 0.22^{a}
	B	0.6	CNB00360	0.07 ± 0.09^{ab}	0.19 ± 0.23^{ab}	0.21 ± 0.23^{ab}	0.07 ± 0.17^{b}	0.09 ± 0.12^{b}	0.10 ± 0.16^{b}	0.28 ± 0.22^{a}
	D	41.9	CND06160	0.16 ± 0.14^{ab}	0.05 ± 0.08^{b}	0.16 ± 0.19^{b}	0.14 ± 0.13^{ab}	0.12 ± 0.19^{b}	0.10 ± 0.16^{b}	0.29 ± 0.23^{a}
	H	0.0	CNH00030	0.24 ± 0.21^{a}	0.20 ± 0.24^{abc}	0.20 ± 0.22^{ab}	0.05 ± 0.12^{bc}	0.07 ± 0.12^{abc}	0.06 ± 0.12^{c}	0.23 ± 0.22^{a}
	L	2.2	CNL04620	0.00^{ab}	0.08 ± 0.14^{ab}	0.14 ± 0.18^{ab}	0.01 ± 0.02^{ab}	0.04 ± 0.04^{ab}	0.08 ± 0.13^{b}	0.23 ± 0.22^{a}
MIC on agar	A	4.2	CNA00290	6.33 ± 1.15^{abc}	6.74 ± 0.67^{a}	6.31 ± 1.20^{ac}	5.11 ± 1.17^{cd}	4.93 ± 1.21^{bd}	4.40 ± 1.76^{bd}	6.62 ± 0.95^{a}
	E	7.8	CNE01630	5.75 ± 1.50^{ab}	6.31 ± 1.14^{ab}	6.12 ± 1.32^{ab}	6.07 ± 1.77^{ab}	5.00 ± 1.15^{ab}	5.13 ± 1.77^{b}	6.15 ± 1.27^{a}
	N	15.0	CNN02060	5.67 ± 1.41^{ab}	5.46 ± 1.66^{ab}	6.43 ± 1.10^{b}	5.50 ± 2.12^{ab}	5.40 ± 1.78^{ab}	5.89 ± 1.36^{ab}	5.35 ± 1.81^{a}
MIC in Broth	A	4.2	CNA00290	6.67 ± 0.58^{a}	4.61 ± 1.24^{a}	5.69 ± 1.14^{a}	3.00 ± 2.55^{abc}	2.56 ± 3.00^{b}	1.00 ± 2.34^{c}	4.63 ± 2.17^{a}
	A	59.8	CNA07470	4.45 ± 1.97^{ab}	4.00 ± 2.65^{a}	5.69 ± 1.14^{a}	3.70 ± 2.54^{ab}	2.04 ± 2.42^{bc}	1.00 ± 2.34^{c}	4.79 ± 2.23^{a}
	C	9.4	CNC06110	1.60 ± 1.95^{a}	1.80 ± 1.92^{a}	4.08 ± 2.44^{a}	3.33 ± 2.66^{a}	4.00 ± 4.12^{a}	2.73 ± 2.88^{a}	4.10 ± 2.65^{a}

Superscript letters refer to significant differences between groups based on Tukey's HSD at $p<0.05$
